# Reviews

**Published:** 1880-04

**Authors:** 


### Reviews.

**Atlas of Human Anatomy.** Containing 180 large plates, arranged according to Drs. Oesterreicher and Erdl, from their original designs from nature, &c., &c., with full and explanatory texts. To be complete in 45 parts, at 75 cents each. By J A. Jeancon, M. D Cincinnati, Ohio: A E. Wilde & Co., publishers

In a former number of the Journal we have directed attention to this admirable work. We have received Parts II, III and IV, and the commendation bestowed upon the first number, and the assurances given by the enterprising publishers that all subsequent numbers would be as complete and perfect as it was possible to make them, have been fulfilled.

The object is to bring before the profession a representation of all parts of the human body, in a size and form which ordinary works on anatomy fail to furnish. It is also purposed to publish as many and as correct microscopic figures bearing upon subjects connected with microscopic anatomy, histology and embryology, as are at present comprised within the scope of medical instruction.

It will be seen that the plan of the work is comprehensive, and if executed, will give the profession one of the most valuable aids to the study of the structure of the human body that has ever been furnished. The price of the atlas is so low that every practitioner and student of medicine should be possessed of it. L.

---

**Brain-work and Over-work.** By Dr. H. C. Wood Philadelphia: Presley Blakiston

One of the American Health Primers, which, as its name indicates, contains a few common sense and plain ideas on the subject of nervous diseases, as augmented by undue mental labor. Trite and generally familiar as is much of the matter, it is presented in a pleasing style, and it would be well if our over-anxious young men, both in professional and mercantile life, would give heed to the warnings in the chapter on work, and to the cxcellent suggestions contained in the succeeding chapters relating to Rest and Recreation. L. H.

